# Metabolic-Associated Fatty Liver Disease and Weight Loss After Bariatric Surgery: A Systematic Review and Meta-Analysis

**DOI:** 10.1007/s11695-024-07585-8

**Published:** 2024-11-19

**Authors:** Fatima Sabench, Elena Cristina Rusu, Helena Clavero-Mestres, Vicente Arredondo-Prats, Marina Veciana-Molins, Sara Muñiz-Piera, Margarita Vives, Carmen Aguilar, Elia Bartra, Marta París-Sans, Ajla Alibalic, Maria Teresa Auguet Quintillà

**Affiliations:** 1 Hospital Universitari Sant Joan de Reus, Reus, Spain; 2 Rovira I Virgili University, Tarragona, Spain; 3 Institut d’Investigació Sanitària Pere Virgili, Tarragona, Spain; 4 Hospital Universitari Joan XXIII de Tarragona, Tarragona, Spain

**Keywords:** Weight loss, NAFLD/MASLD, MASH, Bariatric surgery, Sleeve gastrectomy, Roux-en-Y gastric bypass

## Abstract

**Background:**

Metabolic Dysfunction-Associated Steatotic Liver Disease (MASLD) and Metabolic Dysfunction-Associated Steatohepatitis (MASH) are increasingly prevalent in patients undergoing bariatric surgery (BS). Understanding their impact on weight loss outcomes after surgery and highlighting the results of surgical techniques such as Roux-en-Y Gastric Bypass (RYGB) and Sleeve Gastrectomy (SG) in relation to the presence of MASH are essential for improving patient management and predicting long-term success.

**Methods:**

A systematic review and meta-analysis were conducted. We searched the PubMed database; inclusion criteria were BS patients with liver impairment data at surgery and weight loss data at follow-up of 6 months or longer. Meta-analyses were conducted using R’s meta package, assessing heterogeneity with the *I*^2^ statistic and employing subgroup analyses where necessary.

**Results:**

Out of 1126 eligible studies, 22 were included in the final systematic review. For the MASLD vs. Normal Liver (NL) comparison, no significant difference in BMI change was found at 12 months, but subgroup analysis indicated a possible publication bias (published data vs data collected). In the MASH vs. non-MASH comparison, high heterogeneity was noted at 12 months, and further stratification by surgical technique revealed that SG patients with MASH experienced lower weight loss, approaching statistical significance.

**Conclusions:**

MASLD does not significantly affect short-term weight loss outcomes post-BS, but long-term results show variability. Standardized reporting practices and complete data dissemination are essential for future research to enhance meta-analysis reliability and generalizability.

**Graphical Abstract:**

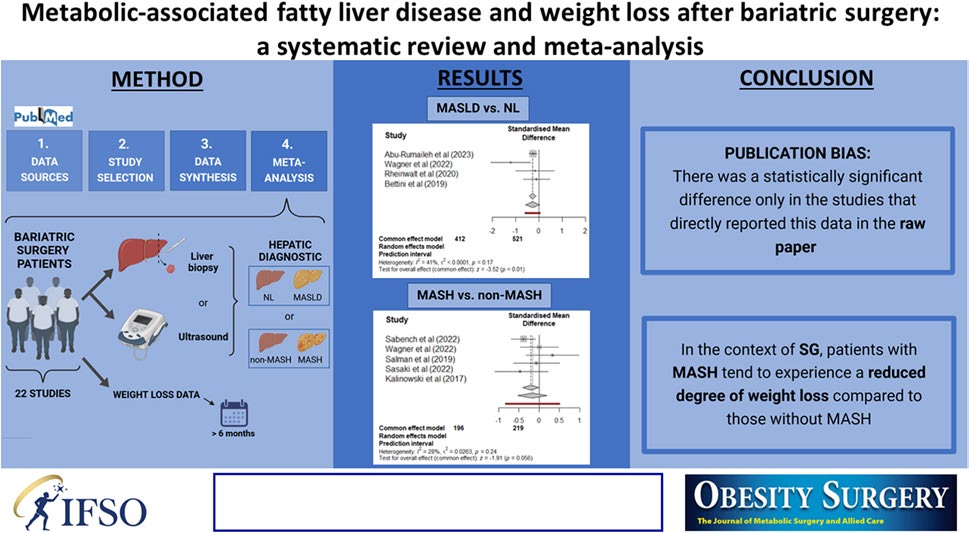

## Introduction

Obesity is a chronic disease defined by a body mass index (BMI) greater than 30 kg/m^2^, and its prevalence is increasing exponentially worldwide, creating a significant global health problem [1]. Classified as a pandemic, it leads to the development of type 2 diabetes mellitus (T2DM), metabolic syndrome, and fatty liver disease, the latter recently renamed as MASLD (Metabolic dysfunction-Associated Steatotic Liver Disease), among other associated problems [2, 3]. MASLD represents a clinical syndrome caused by excessive fat deposition in hepatocytes and includes histopathological entities ranging from simple steatosis (SS) and metabolic dysfunction associated steatohepatitis (MASH) to its advanced stages, including hepatic fibrosis and cirrhosis [4]. It is a highly prevalent disease affecting approximately 25% of the global population but can increase up to 60% in diabetic patients and up to 90% in individuals with severe obesity [5]. Bariatric surgery (BS), in addition to inducing significant weight loss in patients with obesity, has also been associated with histological improvement of SS, MASH, and even partial regression of fibrosis in early cases [6]. Furthermore, BS is also associated with a substantial reduction in the risk of progression from MASH to cirrhosis (88% according to recently published data [7]). However, few studies have analyzed the relationship between weight loss after BS based on the presence and stage of MASLD. A recent study highlights those patients without underlying liver disease lose more weight than those with low- or high-grade liver disease after sleeve gastrectomy (SG) [8]. There have been slight differences in the percentage of excess weight loss (%EWL) 12 months after gastric bypass (RYGB) between patients with MASH and those with SS. While both means exceeded 50% of their value, this does not discriminate between possible differences that may exist due to the bias exerted by the initial BMI value [9]. Weight regain after surgery can worsen MASLD or delay its improvement, and clinically, it is also important to refine the indication of the appropriate surgical technique based on the patient’s liver histology. In this sense, patients with MASLD/MASH could benefit more from techniques that achieve a sustained weight loss pattern, as seen with RYGB [10, 11].

Regarding different indicators of weight loss after BS, %EWL is more sensitive to error than total weight loss (%TWL) and adjustable weight loss (%AWL), as it is closely related to the ideal weight. %TWL is a parameter that should be used in all scientific communications and publications, and although we do not yet have sufficient evidence regarding %AWL, it seems to be even more precise than %TWL [12]. %TWL allows for comparisons between different patient series with less bias from initial BMI, and %AWL is also very useful in cohorts of diabetic patients [13, 14]. A recent study demonstrates that after analyzing weight loss through these indicators, both diabetic patients undergoing RYGB and those undergoing SG showed no differences in weight loss in the presence of MASH. On the other hand, non-diabetic patients undergoing SG lost less weight in the presence of MASH compared to patients without MASH; however, the presence of MASH did not affect weight loss in non-diabetic patients undergoing RYGB. Therefore, there seem to be relevant differences in weight loss patterns between patients with or without MASH, especially after SG [15].

The objective of this meta-analysis is to analyze the effect of the presence of MASLD and MASH on weight loss after BS using the two most frequently used techniques worldwide (SG and RYGB) and based on different weight loss indicators.

## Materials And Methods

### Data Sources

For the purpose of conducting this systematic review and meta-analysis, our main research question was formulated as “Is weight loss after bariatric surgery influenced by the presence of MASLD/ MASH?” Table 1 depicts the population, intervention, comparator, and outcomes (PICO) structure of information. The systematic review was registered at PROSPERO (https://www.crd.york.ac.uk/prospero/).

**Table 1:** Researchable question definition via the PICO structure

Population	Human subjects of any age and any degree of obesity (but having obesity) undergoing bariatric surgery and with liver diagnosis
Intervention	Bariatric surgery, later limited to RYGB and SG
Comparison	Patients with and without MASLD (diagnosed by either liver biopsy, imaging or ultrasound techniques) and patients with and without MASH (diagnosed by liver biopsy)
Outcomes	Weight loss measurements after bariatric surgery; change in BMI, TWL, EWL, and weight at different time points

To fully capture the literature related to this topic, we performed two rounds of searches in the PubMed (https://pubmed.ncbi.nlm.nih.gov/) online database, the first one used the query “((bariatric surgery[Title/Abstract]) OR (Sleeve Gastrectomy[Title/Abstract]) OR (gastric bypass[Title/Abstract])) AND ((liver[Title/Abstract]) OR (nafld[Title/Abstract]) OR (MASH[Title/Abstract]) OR (masld[Title/Abstract]) OR (mash[Title/Abstract])) AND (weight[Title/Abstract])” and included studies from 2018 onwards. The second search used the query “(“bariatric surgery” OR “Sleeve Gastrectomy” OR “gastric bypass” OR “gastric band”) AND (“fatty liver” OR nafld OR MASH OR masld OR mash) AND (weight OR BMI OR EWL OR TWL),” without any date specifications. Both searches included the human and adult filters. In addition, we also inspected other sources, such as references of relevant publications. To avoid publication bias, we intentionally included studies whose main outcome was not the assessment of weight loss after bariatric surgery but reported this data incidentally.

### Study Selection

Study inclusion criteria were bariatric surgery patients, the existence of two groups based on liver impairment, and weight loss data at any follow-up 6 months or longer after the surgery. Exclusion criteria included the lack of follow-up weight loss data, case reports, reviews or guidelines, animal studies, and fibrosis and cirrhosis comparisons. Given the vast majority of studies that did not provide weight loss data stratified by initial liver status, we opted to contact the authors requesting it. We did not request any data from studies prior to 2014, due to the low likelihood of getting answers.

We did not exclude any papers based on language but rather used the online tool DocTranslator (www.onlinedoctranslator.com) to assess their eligibility.

The quality of the studies was evaluated independently by two authors (V.A. and M.V.) using the scale described by Qumseya [16].

### Data Synthesis

Several studies presented data as medians and quartile ranges. We used the quantile estimation method for estimating the mean and standard deviation using the R (v3.6.1) package estmeansd (v 1.0.1) [17]. For several reports, means and standard deviations had to be combined (for instance, simple steatosis and MASH groups both combined into a MASLD group, which encompassed the broad spectrum of the disease), using the following formulas, where *m* is the mean (_c_ for combined, _1_ and _2_ for the groups to combine), *σ* is the standard deviation, and *n*_1_ and *n*_2_ are the samples sizes for both groups:

$$\begin{array}{cc}{m}_{c}=\frac{{n}_{1}\bullet {m}_{1}+{n}_{2}\bullet {m}_{2}}{{n}_{1}+{n}_{2}}& {\sigma }_{c}=\sqrt{\frac{\left({n}_{1}-1\right)\bullet {\sigma }_{2}^{1}+{n}_{1}\bullet {m}_{1}^{2}+\left({n}_{2}-1\right)\bullet {\sigma }_{2}^{2}+{n}_{2}\bullet {m}_{2}^{2}-\left({n}_{1}+{n}_{2}\right)\bullet {m}_{c}^{2}}{{n}_{1}+{n}_{2}-1}}\end{array}$$

Finally, given that some studies indicated BMI at baseline and at follow-up, but did not include directly the ∆BMI with the corresponding standard deviation, we had to infer the standard deviation. For this, we used the formula described by McNemar [18] with a correlation of 0.6 (estimated from Salman et al.’s [19] raw data).

### Meta-Analysis

Studies were grouped according to liver impairment histological classification ([1] normal liver (NL) group versus MASLD and [2] non-MASH versus MASH), weight measurement, and follow-up time. When follow-up time was not equal for all study participants, the study was assigned to the closest time mark.

We used R’s package meta (v7.0–0, [20]), which calculates common and random effect estimates using inverse variance weighting for pooling. Effect sizes were assessed with forest plots, and study heterogeneity was evaluated with the *I*^2^ statistic and funnel plots. The random effects model was used when *I*^2^ > 0.05; otherwise, the common effect model was applied. Sub-group analyses were performed in order to find the source of heterogeneity in instances when *I*^2^ > 0.05. *P*-values < 0.05 were considered statistically significant.

Variables used for sub-group analyses included data source (original report or email), surgery type, a statistically significant difference in age or initial BMI between groups, or more than 15% difference in sex distribution between groups.

## Results

### Identified Records

We retrieved a total of 1126 eligible studies; 1032 records were screened and 247 were assessed for eligibility. From these, sixteen studies provided the data in the paper or supplementary materials, while 140 indicated that the data was available but did not include it in the original paper (generally, weight loss data was not stratified by liver status at baseline). Out of the 108 emails sent to the authors, responses were received for 13, seven of them provided the data, and finally, six studies were included (one study [21] was discarded due to having classified patients only according to NAFLD Fibrosis Score, NFS).

**Fig. 1:**
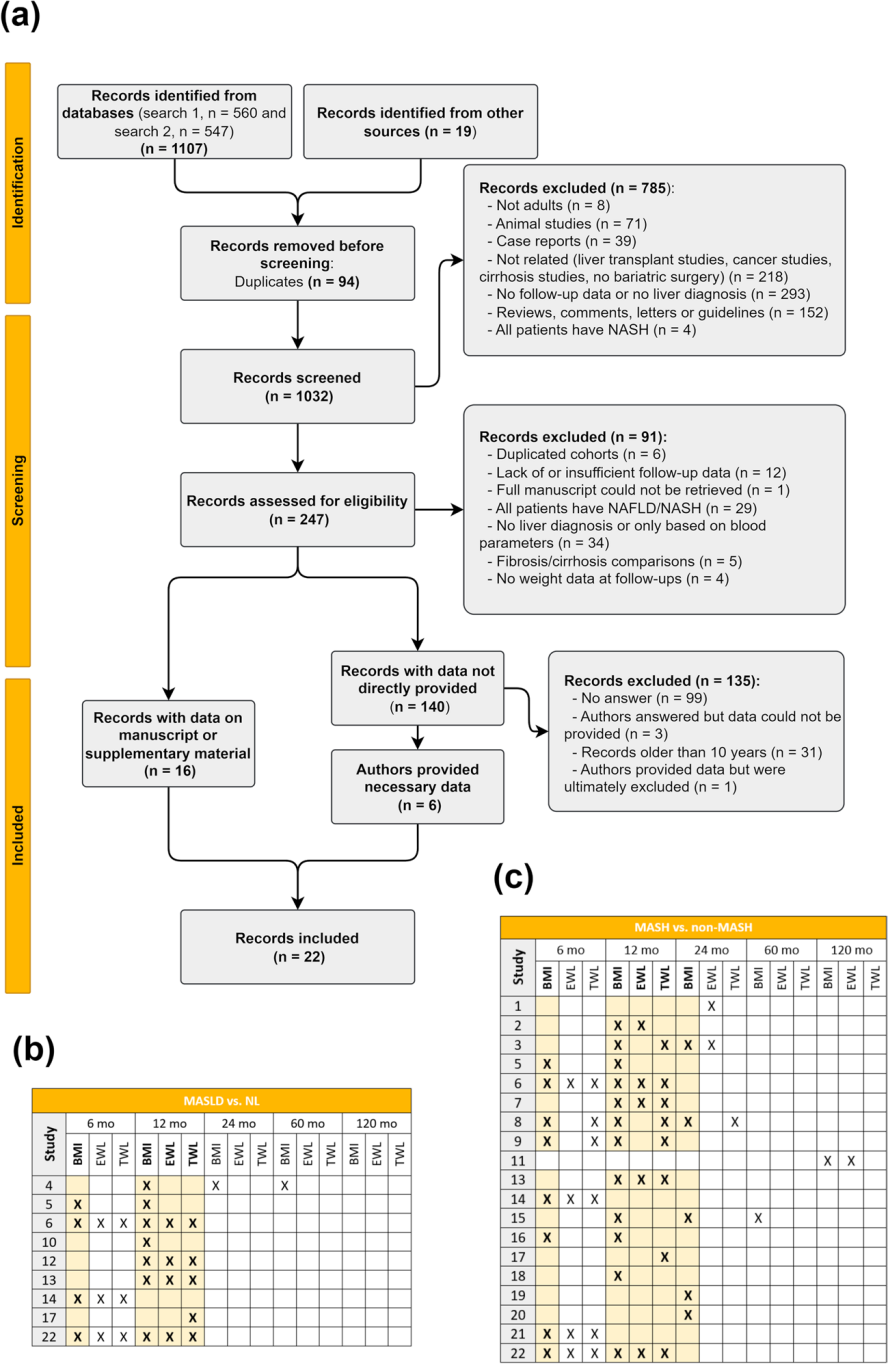
Study selection and grouping. **a** PRISMA diagram for the studies included in the systematic review, with exclusion reasons. **b** Studies included in the MASLD vs. NL comparison; **c** studies included in MASH vs. non-MASH comparison. Boldface highlighted cells mark the weight measurement and time points analyzed

As depicted in Fig. 1A, a total of 22 studies were included in the final meta-analysis. We assessed the possible study combinations regarding liver histological classification, weight measurement, and time points. For the MASLD vs. NL comparison (Fig. 1B), sufficient records were found for the change in BMI at 6 and 12 months, as well as for EWL and TWL at 12 months. For the MASH vs. non-MASH comparison (Fig. 1C), the change in BMI can be assessed at 6, 12, and 24 months, and EWL and TWL can be evaluated at 12 months. No other weight loss measurements were widely available in the screened reports. Table 2 reports all specifications from the included studies.

**Table 2:** Demographic data for all studies included

Study	Reference	n	Liver diagnosis	Classification	Comparison	Bariatric Surgery*	Country	Data source	Significantly different age	Significantly different BMI	> 15% difference in sex	Evaluation
1	Abdalla T. S. A. (2023) [22]	288	Liver biopsy	NAS	MASH vs. non-MASH	SG, RYGB	Germany	1	NA	No	NA	6 (6, 6)
2	Abbassi Z. (2022) [9]	491	Liver biopsy	SAF score	MASH vs. non-MASH	RYGB	Switzerland	1	Yes	No	Yes	7 (7, 7)
3	Sabench F. (2022) [15]	410	Liver biopsy	Brunt	MASH vs. non-MASH	SG, RYGB	Spain	1	No	No	No	8 (8, 8)
4	Abu-Rumaileh M. (2023) [23]	714	Liver biopsy, Imaging studies	NFS, EMERSE	MASLD vs. NL	SG, RYGB	USA	2	No	No	No	5 (5, 5)
5	Wagner K. T. (2022) [8]	77	Liver biopsy	METAVIR classification	MASH vs. non-MASH	SG	USA	1	NA	No	NA	6.5 (7, 6)
					MASLD vs. NL				NA	No	NA	6.5 (7, 6)
6	Rheinwalt K. P. (2020) [24]	143	Liver biopsy	NAS	MASH vs. non-MASH	RYGB, OAGB	Germany	1	No	No	No	7 (7, 7)
					MASLD vs. NL				No	Yes	No	7 (7, 7)
7	Salman M. A. (2020) [19]	94	Liver biopsy	NAS	MASH vs. non-MASH	SG	Italy	1	No	No	No	5.5 (6, 5)
8	Nikai H. (2020) [25]	68	Liver biopsy	NAS, Brunt	MASH vs. non-MASH	SG	Japan	1	No	Yes	Yes	5 (5, 5)
9	Sasaki A. (2022) [26]	63	Liver biopsy	FLIP algorithm, NAS, Matteoni, Brunt	MASH vs. non-MASH	SG	Japan	1	No	No	No	5.5 (6, 5)
10	Bettini S. (2019) [27]	56	Ultrasound	Steatosis presence or absence	MASLD vs. NL	SG	Italy	1	No	No	Yes	7 (7, 7)
11	Tan C. H. (2019) [28]	231	Liver biopsy	NAS	MASH vs. non-MASH	OAGB, LAGB, SG	Taiwan	1	No	No	No	4.5 (5, 4)
12	Khalaj A. (2020) [29]	800	Ultrasound	Based on echogenicity	MASLD vs. NL	RYGB, OAGB	Iran	2	NA	NA	NA	6.5 (6, 7)
13	Blume C. A. (2021) [30]	461	Liver biopsy, Ultrasound	Kleiner	MASH vs. non-MASH	RYGB	Brasil	2	NA	NA	NA	5 (4, 6)
					MASLD vs. NL				NA	NA	NA	5 (4, 6)
14	Perdomo C. M. (2021) [31]	89	Liver biopsy	Kleiner	MASH vs. non-MASH	SG, RYGB	Spain	2	No	No	Yes	6.5 (6, 7)
					MASLD vs. NL				No	No	Yes	6.5 (6, 7)
15	Uehara D. (2019) [32]	84	Liver biopsy	NAS, Brunt	MASH vs. non-MASH	SG, RYGB	Japan	1	NA	No	NA	5.5 (7, 4)
16	Kalinowski P. (2017) [10]	66	Liver biopsy	NAS, Brunt	MASH vs. non-MASH	SG, RYGB	Poland	1	Yes	No	Yes	7.5 (8, 7)
17	Ooi G. J. (2017) [33]	84	Liver biopsy	NAS, Kleiner	MASH vs. non-MASH	LAGB	Nicaragua	1	No	No	No	6 (6, 6)
					MASLD vs. NL				No	No	No	6 (6, 6)
18	Anjani K. (2015) [34]	46	Liver biopsy	SAF score	MASH vs. non-MASH	RYGB	France	1	Yes	Yes	No	7 (7, 7)
19	Felipo V. (2013) [35]	47	Liver biopsy	NAFLD scoring system	MASH vs. non-MASH	RYGB	Spain	1	No	No	No	6.5 (6, 7)
20	Chisholm J. (2012) [36]	9	Liver biopsy	Brunt	MASH vs. non-MASH	LAGB	Australia	1	No	NA	No	6.5 (6, 7)
21	Takahashi N. (2022) [37]	20	Liver biopsy	NAS, Brunt	MASH vs. non-MASH	SG	France	2	No	No	No	5.5 (5, 6)
22	Giraudi P.J. (2020) [38]	71	Liver biopsy	NAS, Kleiner	MASH vs. non-MASH	SG, RYGB	Italy	2	NA	No	NA	6 (6, 6)
					MASLD vs. NL				NA	No	NA	6 (6, 6)

*NA*, missing value; 1, paper or supplementary material; 2, email

^*^The analysis of the type of bariatric surgery is limited to RYGB and SG

### MASLD Impact on Weight Loss After Bariatric Surgery

**Fig. 2:**
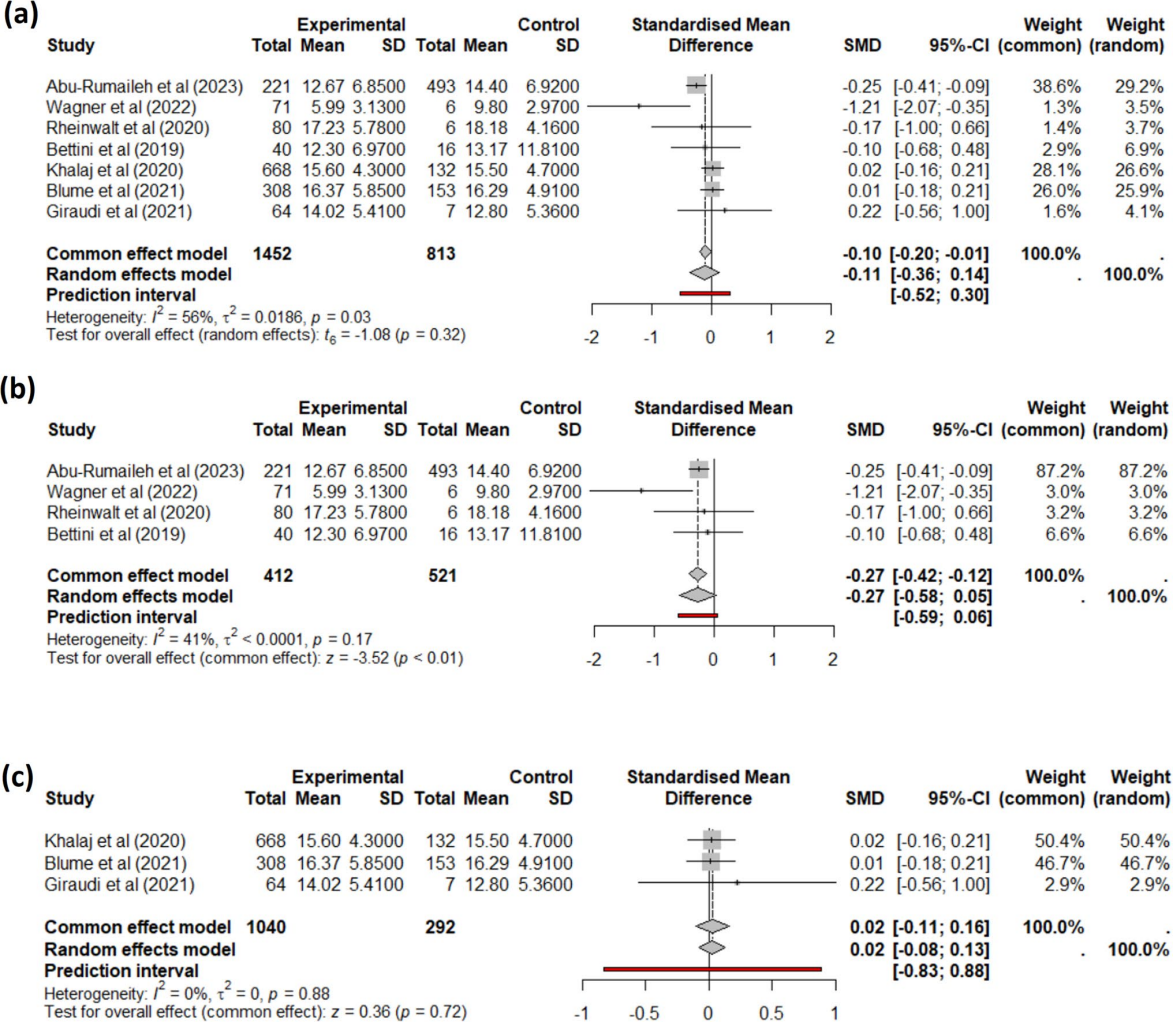
Meta-analysis for the difference in BMI between baseline and 1 year after bariatric surgery for MASLD (experimental group) vs. NL (control group). **a** Combined results and **b** papers describing this data in the publications or supplementary materials; **c** authors provided data a *posteriori*

First, we aimed to evaluate the impact of MASLD presence on weight loss after bariatric surgery. As described in Fig. 1b, we performed a meta-analysis for BMI change at 6 and 12 months, and for EWL and TWL at 12 months. The four studies reporting a change in BMI at 6 months had low heterogeneity (*I*^2^ = 0%) and showed no statistically significant difference in weight loss patterns between patients with MASLD and with NL. Regarding the change in BMI 1 year after surgery, the seven studies were heterogeneous (Fig. 2a); the sub-group analysis based on data source showed that there was a statistically significant difference only in the studies that directly reported this data in the raw paper (Fig. 2b), but this could not be found in the papers that did not directly report this data (Fig. 2c). Regarding EWL and TWL at 12 months, we did not find any difference.

### MASH Impact on Weight Loss After Bariatric Surgery

**Fig. 3:**
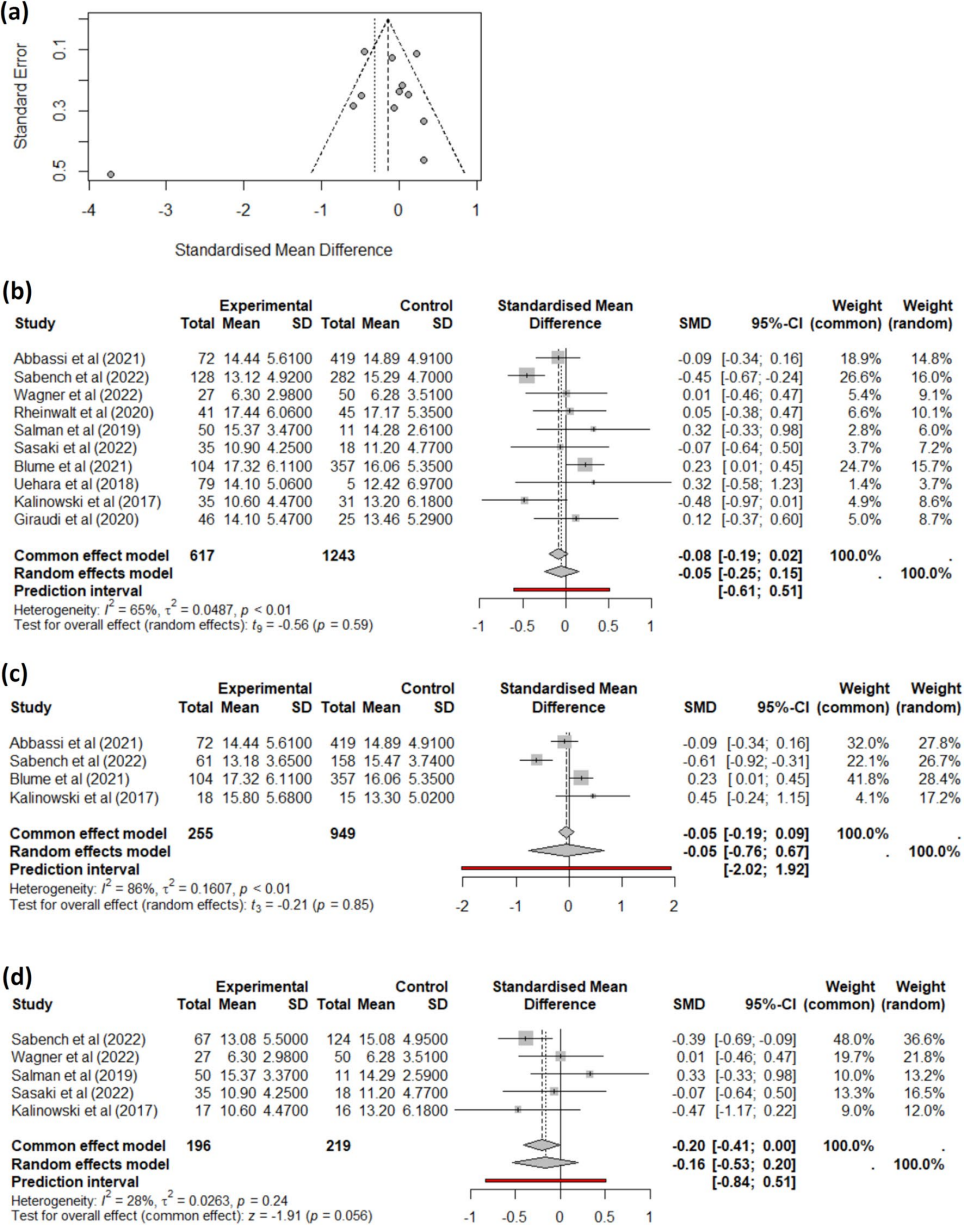
Meta-analysis for the difference in BMI between baseline and 1 year after bariatric surgery for MASH (experimental group) vs. non-MASH (control group). **a** Combined results, including studies where initial BMI is significantly different between groups; **b** meta-analysis of studies with no significantly different initial BMI; **c** meta-analysis of only RYGB studies; and **d** meta-analysis of only SG studies

On the one hand, studies reporting changes in BMI at 6 months had a low heterogeneity, and we did not find any impact of MASH presence on BMI loss at this time point. On the other hand, studies reporting a change in BMI at 1 year initially showed no difference and exhibited high heterogeneity (Fig. 3a). Specifically, the study by Anjani et al. [34] markedly differs from others. Given that the MASH patients from this study had significantly different initial BMI compared to the non-MASH patients, we excluded studies with this circumstance. The remaining ten studies displayed high heterogeneity (Fig. 3b), and the data source did not explain the cause of this heterogeneity. Therefore, we opted to perform sub-group analyses separately for the two main bariatric surgeries: RYGB (Fig. 3c) and SG (Fig. 3d). In this case, a different trend is observed with the SG group’s studies, approaching statistical significance in the context of MASH. At 1 year, EWL and TWL were not statistically different between MASH and non-MASH patients.

**Fig. 4:**
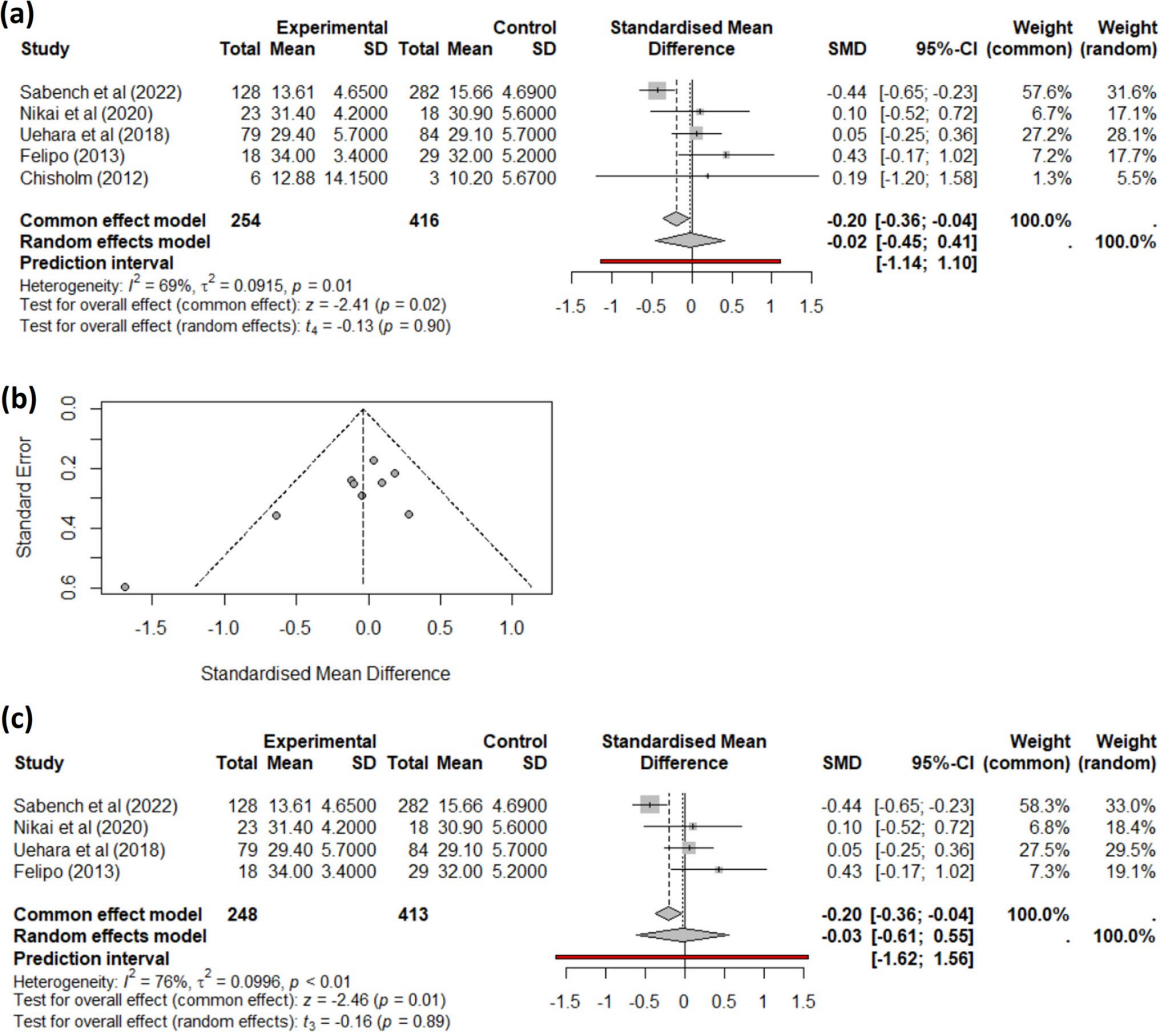
Meta-analysis for the difference in BMI between baseline and 2 years after bariatric surgery for MASH (Experimental group) vs. non-MASH (Control group). **a** Combined results; **b** funnel plot showing study heterogeneity; and **c** meta-analysis of studies with patients undergoing either SG or RYGB

Finally, we assessed the change in BMI 2 years after the bariatric surgery for all studies reporting this data (Fig. 4a). The heterogeneity in these studies was very high, and the funnel plot (Fig. 4b) showed that the study by Chisholm et al. [36] differed from the others, being the only one including patients who undergone LAGB. Therefore, we chose to remove it and assess only studies including SG and RYGB patients, obtaining no difference in BMI change at 24 months after bariatric surgery (Fig. 4c) between patients with MASH and those without the condition. We could not perform sub-group analysis based on surgery type, as there were not enough studies reporting data separately.

## Discussion

Individual studies on weight loss after bariatric surgery in patients with MASLD may yield inconsistent or contradictory results due to differences in the types of bariatric surgeries, the severity of MASLD, and the patient’s characteristics (age, gender, comorbidities, etc.). The results could have important implications for clinical decision-making, as if patients with severe hepatic impairment tend to lose less weight, it may be necessary to adapt pre- and postoperative strategies to optimize their outcomes or refine the indication for the main surgical techniques. It can also help to inform patients about what to expect in terms of long-term outcomes. This has been the hypothesis that has guided the need to conduct a meta-analysis on the subject.

Focusing on the discussion of the results, the absence of significant differences in BMI changes at 6 months suggests that MASLD does not impact early post-surgical weight loss. However, the high heterogeneity and variability in BMI changes observed at 12 months indicate that long-term outcomes could be affected by various factors related to the methodologies used in the analyzed studies, including study design, data reporting, and patient characteristics. That is why we contacted numerous authors, and from the data received, it was observed that there is a clear publication bias in this regard. The manner in which data is reported can substantially affect the perceived efficacy of bariatric surgery in terms of changes in BMI. Specifically, variations in reporting practices—including the statistical measures used (e.g., mean versus median BMI changes), follow-up durations, sample characteristics, definitions of success, and publication biases—can all influence the interpretation of surgical outcomes. The same issues are observed in other studies that analyze the impact of revisional bariatric surgery on weight loss [39]. Examining exclusively the published data for patients with and without MASLD, we observe that individuals with preexisting MASLD experienced significantly less total and excess weight loss compared to those without MASLD. This differential outcome was consistent across both surgical techniques employed (RYGB and SG) [8, 23]. The impact of MASH on weight loss after bariatric surgery was also analyzed, focusing on BMI changes at different time points. There was no significant impact of MASH on the amount of BMI lost at 6 months. The studies reporting BMI changes at 6 months exhibited low heterogeneity, indicating that the results were relatively consistent across these studies. This suggests that, in the short term, the presence of MASH does not affect the weight loss outcomes following bariatric surgery. However, studies reporting BMI changes at 1 year showed high heterogeneity. Despite excluding studies with significant initial BMI differences between groups, the remaining ten studies still displayed high heterogeneity. Despite these results, one of the studies that could not be included in the meta-analysis due to the available follow-up time concluded that patients with MASH showed less weight loss starting at 24 months after surgery [22]. However, another study found no significant differences in weight loss between the MASH and non-MASH groups at a 10-year follow-up [28]. This ongoing variability suggests that other factors, such as the type of surgical technique performed, may influence the results. In this context, when stratifying the studies according to the surgical technique, it is observed that, in the case of SG, patients with MASH experienced a lower degree of weight loss very close to statistical significance, compared to those without MASH. This is consistent with the results found in our recently published patient series [15]. But histopathologically, no significant differences have been observed between SG and RYGB after surgery in a published meta-analysis with controlled studies, which seems to indicate that both procedures may be equally effective in the management of MASH [11]. This raises the question of whether the difference in weight loss can largely be attributed to the surgical technique used. Undoubtedly, having more data would help us confirm or refute this hypothesis SG, also seems to be safer than other procedures in patients with cirrhosis [40]. This can contribute to an increase in the number of SG performed as shown in the IFSO surveys [41], and because of this, the weight results could be not so optimal. We do not know what will happen to liver function in the long term. Recent studies have determined that bariatric surgery reduces the risk of non-alcoholic cirrhosis and liver cancer but may also increase the risk of postoperative alcoholic cirrhosis [42]. This fact is worrying because it tells us a lot about the role that addictions can play after bariatric surgery [43]; furthermore, it may play a determining role in long-term weight loss, which should be also taken into account in future studies on these patients.

It's imporant to emphasize that this analysis involved screening 1126 studies, with 1032 records reviewed and 247 assessed for eligibility. Despite identifying 16 studies with the necessary data, 140 studies lacked data stratified by liver status at baseline, revealing a common issue of incomplete data reporting. Efforts to contact original authors resulted in a low response rate (12%), with only 13 out of 108 authors responding and just seven providing the needed data. These challenges highlight the need for standardized reporting practices and better data sharing to enhance the quality of systematic reviews and meta-analyses [22].

### Limitations

Several studies report significant differences in age or BMI between groups. This variability could affect the generalizability of findings and emphasizes the need to control for these factors in analyses. Additionally, differences in sex distribution (> 15% difference) in some studies might influence weight loss outcomes, as gender can affect metabolic results and weight loss patterns. Most studies used liver biopsy for diagnosis that supports comparability, but different classifications used (NAS, METAVIR, and SAF Score) might influence the results. The limited number of studies could also contribute to variability and heterogeneity in the results.

## Conclusions

While MASLD does not appear to significantly affect short-term weight loss outcomes, there is notable variability in long-term results, emphasizing the need for standardized reporting practices in future research. Specifically, in the context of SG, patients with MASH tend to experience a reduced degree of weight loss compared to those without MASH. Future studies should focus on complete and transparent data reporting to facilitate meta-analyses and systematic reviews. Establishing a culture of cooperation and transparency within the research community is crucial for enhancing the quality and credibility of results, leading to more accurate and generalizable conclusions.

## Data Availability

The data supporting the findings of this study are available within the publication and upon reasonable request to the corresponding author.

## References

[1] Kleiner DE, Brunt EM, Van Natta M, et al. Design and validation of a histological scoring system for nonalcoholic fatty liver disease. Hepatology. 2005;41(6):1313–21. 15915461 10.1002/hep.20701

[2] Younossi Z, Anstee QM, Marietti M, et al. Global burden of NAFLD and NASH: trends, predictions, risk factors and prevention. Nat Rev Gastroenterol Hepatol. 2018;15(1):11–20. 28930295 10.1038/nrgastro.2017.109

[3] Eslam M, Sanyal AJ, George J, et al. MAFLD: a consensus-driven proposed nomenclature for metabolic associated fatty liver disease. Gastroenterology. 2020;158(7):1999-2014.e1. 32044314 10.1053/j.gastro.2019.11.312

[4] Lassailly G, Caiazzo R, Ntandja-Wandji LC, et al. Bariatric surgery provides long-term resolution of nonalcoholic steatohepatitis and regression of fibrosis. Gastroenterology. 2020;159(4):1290-1301.e5. 32553765 10.1053/j.gastro.2020.06.006

[5] Polyzos SA, Kountouras J, Mantzoros CS. Obesity and nonalcoholic fatty liver disease: from pathophysiology to therapeutics. Metabolism. 2019;92:82–97. 30502373 10.1016/j.metabol.2018.11.014

[6] Machado M, Cortez-Pinto H. Diet, microbiota, obesity, and NAFLD: a dangerous quartet. Int J Mol Sci. 2016;17(4):481. 27043550 10.3390/ijms17040481 PMC4848937

[7] Aminian A, Al-Kurd A, Wilson R, et al. Association of bariatric surgery with major adverse liver and cardiovascular outcomes in patients with biopsy-proven nonalcoholic steatohepatitis. JAMA. 2021;326(20):2031. 34762106 10.1001/jama.2021.19569 PMC8587225

[8] Wagner KT, Randall JA, Zimmermann J, et al. Effects of liver pathology on sleeve gastrectomy outcomes. J Laparoendosc Adv Surg Tech. 2022;32(3):310–4. 10.1089/lap.2021.0799 35021881

[9] Abbassi Z, Orci L, Meyer J, et al. Impact of nonalcoholic steatohepatitis on the outcome of patients undergoing roux-en-y gastric bypass surgery: a propensity score–matched analysis. Obes Surg. 2022;32(1):74–81. 34546514 10.1007/s11695-021-05642-0 PMC8752524

[10] Kalinowski P, Paluszkiewicz R, Ziarkiewicz-Wróblewska B, et al. Liver function in patients with nonalcoholic fatty liver disease randomized to Roux-en-Y gastric bypass versus sleeve gastrectomy: a secondary analysis of a randomized clinical trial. Ann Surg. 2017;266(5):738–45. 28767558 10.1097/SLA.0000000000002397

[11] De Brito E, Silva MB, Tustumi F, et al. Gastric bypass compared with sleeve gastrectomy for nonalcoholic fatty liver disease: a systematic review and meta-analysis. Obes Surg. 2021;31(6):2762–72. 33846949 10.1007/s11695-021-05412-y

[12] Beisani M, SabenchPereferrer F, Vilallonga R, González López Ó, Molina López A, Del Castillo DD, et al. Seeking an initial-weight-independent metric in a mediterranean cohort of gastric bypass patients: the %AWL revisited. Obes Surg. 2021;31(4):1524–32. 33398625 10.1007/s11695-020-05154-3

[13] Van De Laar AW, Emous M, Hazebroek EJ, et al. Reporting weight loss 2021: position statement of the dutch society for metabolic and bariatric surgery (DSMBS). Obes Surg. 2021;31(10):4607–11. 34283377 10.1007/s11695-021-05580-x

[14] Van De Laar AW, Van Rijswijk AS, Kakar H, et al. Sensitivity and specificity of 50% excess weight loss (50%EWL) and twelve other bariatric criteria for weight loss success. Obes Surg. 2018;28(8):2297–304. 29484610 10.1007/s11695-018-3173-4

[15] Sabench F, Bertran L, Vives M, et al. NASH presence is associated with a lower weight loss one and 2 years after bariatric surgery in women with severe obesity. Obes Surg. 2022;32(10):3313–23. 35792995 10.1007/s11695-022-06175-w

[16] Qumseya BJ. Quality assessment for systematic reviews and meta-analyses of cohort studies. Gastrointest Endosc. 2021;93(2):486-494.e1. 33068610 10.1016/j.gie.2020.10.007

[17] McGrath S, Zhao X, Steele R. Estimating the sample mean and standard deviation from commonly reported quantiles in meta-analysis. R package version 0.1.0,. 2019; Available from: https://CRAN.R-project.org/package=estmeansd 10.1177/0962280219889080 PMC7390706 32292115

[18] McNemar Q. Note on the sampling error of the difference between correlated proportions or percentages. Psychometrika. 1947;12(2):153–7. 20254758 10.1007/BF02295996

[19] Salman MA, Salman AA, Abdelsalam A, et al. Laparoscopic sleeve gastrectomy on the horizon as a promising treatment modality for NAFLD. Obes Surg. 2020;30(1):87–95. 31372873 10.1007/s11695-019-04118-6

[20] Balduzzi S, Rücker G, Schwarzer G. How to perform a meta-analysis with R: a practical tutorial. Evid Based Ment Health. 2019;22(4):153–60. 31563865 10.1136/ebmental-2019-300117 PMC10231495

[21] Hempel F, Roderfeld M, Müntnich LJ, et al. Caspase-cleaved keratin 18 measurements identified ongoing liver injury after bariatric surgery. J Clin Med. 2021;10(6):1233. 33809676 10.3390/jcm10061233 PMC8002276

[22] Abdalla TSA, Giannou AD, Abdalla ASA, et al. The effect of non-alcoholic fatty liver disease on weight loss and resolution of obesity-related disorders after bariatric surgery. World J Surg. 2023;47(12):3281–8. 37747548 10.1007/s00268-023-07153-8 PMC10694115

[23] Abu-Rumaileh M, Haddad RA, Yosef M, et al. Impact of nonalcoholic fatty liver disease (NAFLD) on weight loss after bariatric surgery. Obes Surg. 2023;33(12):3814–28. 37940737 10.1007/s11695-023-06865-z PMC12330159

[24] Rheinwalt KP, Drebber U, Schierwagen R, et al. Baseline presence of NAFLD predicts weight loss after gastric bypass surgery for morbid obesity. J Clin Med. 2020;9(11):3430. 33114543 10.3390/jcm9113430 PMC7693802

[25] Nikai H, Ishida K, Umemura A, et al. Effects of laparoscopic sleeve gastrectomy on non-alcoholic steatohepatitis and liver fibrosis in Japanese patients with severe obesity. Obes Surg. 2020;30(7):2579–87. 32124215 10.1007/s11695-020-04515-2

[26] Sasaki A, Umemura A, Ishida K, et al. The concept of indeterminable NASH inducted by preoperative diet and metabolic surgery: analyses of histopathological and clinical features. Biomedicines. 2022;10(2):453. 35203662 10.3390/biomedicines10020453 PMC8962337

[27] Bettini S, Bordigato E, Milan G, et al. SCCA-IgM as a potential biomarker of non-alcoholic fatty liver disease in patients with obesity, prediabetes and diabetes undergoing sleeve gastrectomy. Obes Facts. 2019;12 3:291–306. 10.1159/000499717 PMC6696770 31104052

[28] Tan CH, Al-Kalifah N, Lee WJ, et al. HSCRP as surrogate marker in predicting long term effect of bariatric surgery on resolution of non-alcoholic steatohepatitis. Asian J Surg. 2019;42(1):203–8. 29804707 10.1016/j.asjsur.2018.04.010

[29] Khalaj A, Mousapour P, Motamedi MAK, et al. Comparing the efficacy and safety of Roux-en-Y gastric bypass with one-anastomosis gastric bypass with a biliopancreatic limb of 200 or 160 cm: 1-year results of the tehran obesity treatment study (TOTS). Obes Surg. 2020;30(9):3528–35. 32405910 10.1007/s11695-020-04681-3

[30] Blume CA, Brust-Renck PG, Rocha MK, et al. Development and validation of a predictive model of success in bariatric surgery. Obes Surg. 2021;31(3):1030–7. 33190175 10.1007/s11695-020-05103-0 PMC7666615

[31] Perdomo CM, Gómez-Ambrosi J, Becerril S, et al. Role of ANGPTL8 in NAFLD improvement after bariatric surgery in experimental and human obesity. Int J Mol Sci. 2021;22(23):12945. 34884755 10.3390/ijms222312945 PMC8657645

[32] Uehara D, Seki Y, Kakizaki S, et al. Long-term results of bariatric surgery for non-alcoholic fatty liver disease/non-alcoholic steatohepatitis treatment in morbidly obese Japanese patients. Obes Surg. 2019;29(4):1195–201. 30542827 10.1007/s11695-018-03641-2

[33] Ooi GJ, Burton PR, Doyle L, et al. Effects of bariatric surgery on liver function tests in patients with nonalcoholic fatty liver disease. Obes Surg. 2017;27(6):1533–42. 27966066 10.1007/s11695-016-2482-8

[34] Anjani K, Lhomme M, Sokolovska N, et al. Circulating phospholipid profiling identifies portal contribution to NASH signature in obesity. J Hepatol. 2015;62(4):905–12. 25450212 10.1016/j.jhep.2014.11.002

[35] Felipo V, Urios A, García-Torres ML, et al. Alterations in adipocytokines and cGMP homeostasis in morbid obesity patients reverse after bariatric surgery. Obesity. 2013;21(2):229–37. 23404955 10.1002/oby.20008

[36] Chisholm J, Seki Y, Toouli J, et al. Serologic predictors of nonalcoholic steatohepatitis in a population undergoing bariatric surgery. Surg Obes Relat Dis. 2012;8(4):416–22. 21865094 10.1016/j.soard.2011.06.010

[37] Takahashi N, Sasaki A, Umemura A, et al. Identification of a fatty acid for diagnosing non-alcoholic steatohepatitis in patients with severe obesity undergoing metabolic surgery. Biomedicines. 2022;10(11):2920. 36428489 10.3390/biomedicines10112920 PMC9687903

[38] Giraudi PJ, Giuricin M, Bonazza D, et al. Modifications of IGF2 and EGFR plasma protein concentrations in NAFLD patients after bariatric surgery. Int J Obes. 2021;45(2):374–82. 10.1038/s41366-020-00687-0 32943763

[39] Bastos ELS, Salgado W, Dantas ACB, et al. Medium and long-term weight loss after revisional bariatric surgery: a systematic review and meta-analysis. Obes Surg. 2024;34(5):1917–28. 38573390 10.1007/s11695-024-07206-4

[40] Bai J, Jia Z, Chen Y, et al. Bariatric surgery is effective and safe for obese patients with compensated cirrhosis: a systematic review and meta-analysis. World J Surg. 2022;46(5):1122–33. 35275232 10.1007/s00268-021-06382-z

[41] Angrisani L, Santonicola A, Iovino P, et al. IFSO worldwide survey 2020–2021: current trends for bariatric and metabolic procedures. Obes Surg. 2024;34(4):1075–85. 38438667 10.1007/s11695-024-07118-3 PMC11026210

[42] Wang G, Huang Y, Yang H, et al. Impacts of bariatric surgery on adverse liver outcomes: a systematic review and meta-analysis. Surg Obes Relat Dis. 2023;19(7):717–26. 36890087 10.1016/j.soard.2022.12.025

[43] Riedel O, Braitmaier M, Dankhoff M, et al. Alcohol use disorders after bariatric surgery: a study using linked health claims and survey data. Int J Obes [Internet]. 2024 Sep 6 [cited 2024 Oct 18]; Available from: https://www.nature.com/articles/s41366-024-01606-3 10.1038/s41366-024-01606-3 PMC11502494 39242916

